# Clinical applications of PD-L1 bioassays for cancer immunotherapy

**DOI:** 10.1186/s13045-017-0479-y

**Published:** 2017-05-17

**Authors:** Delong Liu, Shuhang Wang, Wendy Bindeman

**Affiliations:** 1 Department of Oncology, The first Affiliated Hospital of Zhengzhou University, Zhengzhou, 450052 China; 2 The Key Laboratory of Carcinogenesis and Translational Research (Ministry of Education), Peking University Cancer Hospital, Beijing, China; 3 Department of Immunology, Mayo Clinic, Rochester, MN 55905 USA

## Abstract

Programmed death ligand 1 (PD-L1) has emerged as a biomarker that can help to predict responses to immunotherapies targeted against PD-L1 and its receptor (PD-1). Companion tests for evaluating PD-L1 expression as a biomarker of response have been developed for many cancer immunotherapy agents. These assays use a variety of detection platforms at different levels (protein, mRNA), employ diverse biopsy and surgical samples, and have disparate positivity cutoff points and scoring systems, all of which complicate the standardization of clinical decision-making. This review summarizes the current understanding and ongoing investigations regarding PD-L1 expression as a potential biomarker for clinical outcomes of anti-PD-1/PD-L1 immunotherapy.

## Background

Novel therapeutics targeting immune checkpoints are leading to a fast and profound paradigm shift in cancer therapy [1–3]. Durable responses to agents targeting programmed cell death-1 protein receptor (PD-1) and the ligand (PD-L1) have been observed in lung cancer as well as a variety of cancer types [4–10]. PD-L1 expression varies due to the dynamic tumor microenvironment [11–15]. No consensus has been reached on whether PD-L1 expression can serve as a reliable biomarker for patient selection in all cancer types. The assessment for PD-L1 expression is becoming routine for many cancer specimens, though how to employ the results remains a clinical challenge. This article reviews existing data of PD-L1 expression status and its predictive and prognostic role in lung cancer and makes recommendations for improving clinical interpretations.

## Bioassays for PD-L1 expression

A companion diagnostic is necessary for the safe and efficacious use of a corresponding drug or biological product, whereas a complementary diagnostic identifies, though not essential, a biomarker that can assist in the risk/benefit assessment for a subset of patients who respond to the corresponding drug differentially [16, 17]. Currently, the US Food and Drug Administration (FDA) has approved four immunohistochemistry (IHC)-based assays using diagnostic monoclonal antibodies, 22C3, 28-8, SP142, and SP263, to detect PD-L1 expression and aid in clinical applications of corresponding drugs (Table 1) [18–20]. 22C3 is the mouse hybridoma clone 22C3 monoclonal antibody (IgG1k) against the extracellular domain of human PD-L1 (Phe19-Thr239) [21]. In Western blotting, the 22C3 antibody labels a 40-kDa protein band which corresponds to the recombinant human PD-L1 protein. 28-8 is a hybridoma clone generated after immunization of rabbits with the extracellular domain of human PD-L1 (Phe19-Thr239) [22]. This clone was screened by IHC using control cell lines with or without expression of huPD-L1 or huPD-L2 and human normal and tumor specimens with and without PD-L1 expression. The stable transfectant monoclonal anti-PD-L1 clone 28-8 was produced for the PD-L1 IHC assay.

**Table 1:** PD-L1 immunohistochemistry assays for clinical application

Agent	Nivolumab	Pembrolizumab	Atezolizumab	Durvalumab
Antibody	28-8	22C3	SP142	SP263
Isotype and host species	Rabbit IgG	Mouse IgG	Rabbit IgG	Rabbit IgG
Binding site	Extracellular	Extracellular	Intracellular	Intracellular
Cell scored	Tumor cell	Tumor cell	Tumor cell	Tumor cell
		Tumor stroma	Immune cell	
Cutoffs	1, 5, or ≥10%	≥50% tumor cell	1, 5, or ≥10%	≥25%
		≥1% stroma		

Notably, PD-L1 IHC 22C3 pharmDx and IHC 28-8 pharmDx are the first two FDA-approved assay systems for qualitative detection of PD-L1 protein in formalin-fixed, paraffin-embedded (FFPE) tissue. The level of PD-L1 protein expression is determined by using tumor proportion score (TPS) [22, 23]. The TPS score is the percentage of tumor cells showing partial or complete membrane staining at any intensity. Positive PD-L1 expression is defined as TPS ≥1%, and a specimen is considered to have high PD-L1 expression if TPS ≥50%.

The third FDA-approved antibody, SP142, is a rabbit monoclonal antibody against PD-L1 [24, 25]. This antibody has been validated and approved for use in the complementary assay kit, Ventana PD-L1 (SP142), to detect PD-L1 expression and guide clinical therapy with atezolizumab for advanced urothelial carcinoma and non-small cell lung cancer (NSCLC) [26–29]. The complementary assay considers PD-L1 expression levels (level 1, 2, or 3) in both tumor cells (TC; 1/2/3) and immune cells (IC; 1/2/3) for positivity. Various combinations of TC and IC expression levels were used to define positivity (cutoff values) in the clinical trials [27, 30–32].

The fourth PD-L1 antibody, SP263, was just approved as a complementary diagnostic for durvalumab. SP263 is a rabbit anti-human PD-L1 monoclonal antibody directed against the cytoplasmic region of human PD-L1 [33] that has been optimized and validated for use with FFPE NSCLC and HNSCC tissue samples. The antibody SP263 is approved for qualitative detection of PD-L1 protein in FFPE NSCLC and other tissues. Clinical cutoff values have been validated in clinical trials for guiding the use of durvalumab (NCT01693562) [18, 34].

Recently, studies have also analyzed the PD-L1 expression at the genetic level. PD-L1 and PD-1 protein expression were analyzed in 94 clinical cases of small cell neuroendocrine carcinomas by IHC using two different monoclonal antibodies (5H1, E1L3N). RNA-seq was used to profile messenger RNA (mRNA) expression in 43 clinical cases. Results showed that RNA-seq yielded highly comparable results with IHC and even more PD-L1-positive cases than IHC; therefore, RNA-seq is also suitable for detection of PD-L1 expression [35]. This study also showed that the carcinoma cells were negative for PD-L1 expression in all cases, and PD-L1 was detected in tumor-infiltrating macrophages and lymphocytes. Another study examined 133 cases of lung adenocarcinoma surgical samples and found that the PD-L1 expression rate was 16.5% at the mRNA level and 13.5% at the protein level [36]. These two levels are highly concordant (Kappa = 0.824), suggesting the feasibility of using mRNA level as a biomarker for PD-L1 expression. Another study analyzed DNA copy number and mRNA expression of PD-L1 in 335 patients with soft tissue sarcomas (STS) by utilizing the sarcoma data set of The Cancer Genome Altas (TCGA) and an independent cohort of untreated high-grade STS [37]. This analysis showed that PD-L1copy number and elevated mRNA expression have prognostic significance.

## PD-L1 expression and cutoff values

So far, analysis of the relationship between PD-L1 expression and response to anti-PD-1/PD-L1 agents has yielded promising results. One pooled analysis summarized data from seven studies with 914 NSCLC patients [38]. Patients with TC staining ≥1% were considered PD-L1-positive. These patients had a significantly higher objective response rate (ORR) than those with PD-L1-negative tumors. Further, the PD-L1 threshold of ≥1% and higher positivity (5 and 50%) has been correlated with responses in a meta-analysis [39]. The higher the PD-L1 expression in the specimens, the higher the clinical ORR to the anti-PD-1/PD-L1 agents. In another meta-analysis of 13 studies with 1979 NSCLC patients, ORR correlation with PD-L1 expression levels assayed with several antibodies (DAKO 28-8, DAKO 22C3, VENTANA SP 142) were compared. The ORR generally increased with the level of PD-L1 expression increasing from 1, 5, 10, 25, 50 to 75% [40]. In summary, currently available data support the hypothesis that tumor PD-L1 positivity is a useful biomarker for predicting patient response to anti-PD-1/PD-L1 agents.

An ongoing challenge to the application of PD-L1 biomarker assays in clinic is that each anti-PD1/PD-L1 agent has its own companion assay. These assays have not been standardized for all the agents and therefore are not interchangeable. However, several groups have examined the degree of agreement between different methods. One large study of 493 samples compared the extent of concordance among three validated, commercially available PD-L1 IHC assays (Ventana SP263, Dako 22C3, and Dako 28-8) for NSCLC patients and found an overall percentage agreement of >90% between assays. These assays were also consistent at multiple expression cutoffs, including 1, 10, 25, and 50% tumor cell membrane staining [41]. Most recently, Gaule et al. assessed PD-L1 expression using six monoclonal antibodies (SP142, E1L3N, 9A11, SP263, 22c3, and 28-8) on a genetically defined PD-L1 engineered cell line array with a range of controlled protein-expressing cell lines. They found that all six antibody reagents had high levels of concordance in this IHC standardization study [42, 43]. This study further suggested that differences in PD-L1 expression in tissues as described in the previous studies were independent of the antibody used. Rather, the differences were attributed to the tumor heterogeneity, assay-, or platform-specific variables.

## Factors affecting bioassays for PD-L1 expression

PD-L1 expression is commonly focal and primarily identified at the tumor–stromal interface [44] and appears to be highly heterogeneous. Additionally, PD-L1 expression detection can be limited by the size and position of the biopsy specimen and therefore provides only a snapshot of the expression status of a tumor.

One study examined PD-L1 expression with the SP142 IHC assay in both whole surgical tissue sections and matched lung biopsies from 160 patients with operable NSCLC. PD-L1 expression was assayed in both TC and IC cells. The study found that PD-L1 expression between the surgically resected and matched biopsy specimens frequently disagree with each other (overall discordance rate = 48%, *κ* = 0.218 [poor agreement]) [45]. The PD-L1 assay underestimated the expression from larger resected tumor specimens. The rate of discordance was inversely proportional to the number of cores obtained. Sampling error is therefore a significant issue in the detection of PD-L1, and a single biopsy specimen with few cores may not accurately reflect the PD-L1 status of a tumor. This report is contradicted by another study which retrospectively compared small biopsy samples with resected specimens from 79 NSCLC patients. This latter study found that the positivity rate of PD-L1 assessed by IHC in the biopsy samples was 38.0 versus 35.4% in the resected specimens. This group found a concordance rate of 92.4% and *κ* value of 0.8366, suggesting that there is good concordance and adequate assessment for PD-L1 expression with small samples [46]. These retrospective studies and others are limited in relatively small sample sizes [45–47].

In addition to the focal, heterogeneous expression of PD-L1 within a single lesion, separate lesions from the same patient may have different intensities and patterns of PD-L1 expression. One study performed quantitative assessment of the heterogeneity of PD-L1 expression in 49 NSCLC whole tissue sections and a corresponding tissue microarray. Two rabbit monoclonal antibodies (E1L3N and SP142) were used for both conventional IHC and quantitative immunofluorescence (QIF). Results showed that assessment of 588 serial section fields of view by QIF exhibited a discordant expression at a frequency of 25% [44].

In addition, PD-L1 expression varies among TC, IC, and immune stroma in a given patient. Using 67 fully resected, multifocal specimens from 32 NSCLC patients to assess intertumoral heterogeneity, Mansfield et al. reported that there was poor agreement of PD-L1 expression between paired lesions of 20 patients by tumor and immune cells [48]. Although expression of PD-L1 is heterogeneous among paired independent lung cancers, this study showed that there are high levels of agreement in intrapulmonary metastasis.

PD-L1 expression in TCs and ICs were examined in a separate study which reported that PD-L1-positive TCs were negatively correlated with PD-L1-positive ICs within tumor stroma. In this analysis of 105 patients with resected stage I pulmonary squamous cell carcinoma, tumor PD-L1 expression and increased CD4+ T cell infiltrations in tumor stroma were found to be independent predictors of better overall survival [49]. Therefore, different components of tumor and immune microenvironment may play variable roles in modulation of responses to cancer immunotherapy.

The heterogeneity also exists among primary, metastatic, and transformed tumors. One study reported a case of NSCLC who was found to have also small cell lung cancer (SCLC) transformation in the lung and liver metastasis at autopsy. The PD-L1 protein was partially expressed in tumor cells with adenocarcinoma histology but not in tumor cells from SCLC transformation [50]. Another study analyzed paired untreated primary lung cancer and metastasis tissues from 98 postmortem cases by microarray to evaluate the heterogeneity of PD-L1 expression and correlated with clinicopathological features [51]. The study found that intratumoral heterogeneity in NSCLC is common (discordance rate 82% between primary and metastatic tissues), while PD-L1 expression was undetectable in both primary and metastatic SCLC tissues.

Taken together, these studies suggest that the results of PD-L1 expression assays are affected by a variety of factors, including specimen size, biopsy location, variable components of tumor and immune microenvironment, and tumor transformation. These factors should be carefully considered when employing PD-L1 as a predictive biomarker in clinical practice.

## Clinical implications of soluble PD-L1

Several members of B7 family have been found to have soluble counterparts [52–54]. Using ELISA, a soluble form of PD-L1 (sPD-L1) has been detected in the sera of patients [55]. The study showed that circulating sPD-L1 in human sera is involved in modulating immune response. It has been further suggested that upregulation of sPD-L1 production is associated with tumor-inspired immune suppression and the poor prognosis [56–58]. In another study of 96 patients with lung cancer (85 NSCLC, 7 SCLC), sPD-L1 was detected by ELISA [59]. High sPD-L1 levels (≥7.32 ng/ml) were associated with poor prognosis (high vs low sPD-L1: OS 13.0 vs 20.4 months, *p* = 0.037) in these patients [59]. It remains unclear whether sPD-L1 level has correlation with clinical response to the checkpoint inhibitor treatment. Liquid biopsy is increasingly used as a substitute of tissue sampling [60–62]. sPD-L1 deserves further investigation to see whether it can be used to guide clinical decisions on choice of immunotherapeutic agents.

## Conclusions

Novel therapeutics targeting immune checkpoints are leading to a fast and profound paradigm shift in cancer therapy. PD-L1 expression is a valuable biomarker to guide clinical decisions. PD-L1 expression assays are affected by a variety of factors, including specimen size, biopsy location, variable components of tumor and immune microenvironment, and tumor transformation. These factors should be carefully considered when employing PD-L1 as a predictive biomarker in clinical practice. Future investigations should focus on standardizing detection, developing reliable methods of liquid biopsy, and developing multiparameter quantitative or semi-quantitative biomarker panels to provide clinicians a more comprehensive understanding of the tumor and immune microenvironment. Correlation of PD-L1 expression and tyrosine kinase biomarkers should also be explored [63].

## Abbreviations

FFPE: Formalin-fixed, paraffin-embedded
IC: Immune cells
IHC: Immunohistochemistry
NSCLC: Non-small cell lung cancer
ORR: Objective response rate
OS: Overall survival
PD-1: Programmed death-1
PD-L1: Programmed death-1 ligand
QIF: Quantitative immunofluorescence
SCLC: Small cell lung cancer
STS: Soft tissue sarcoma
TC: Tumor cells
TCGA: The Cancer Genome Altas
TPS: Tumor proportion score
